# The pathogenicity of avian metapneumovirus subtype C wild bird isolates in domestic turkeys

**DOI:** 10.1186/1743-422X-10-38

**Published:** 2013-01-30

**Authors:** Ra Mi Cha, Qingzhong Yu, Laszlo Zsak

**Affiliations:** 1 Southeast Poultry Research Laboratory, Agricultural Research Service, United States Department of Agriculture, 934 College Station Road, Athens, GA 30605, USA

**Keywords:** Avian metapneumovirus, Wild bird, Pathogenicity, Turkeys

## Abstract

**Background:**

Avian metapneumovirus subtype C (aMPV/C) causes severe upper respiratory disease in turkeys. Previous report revealed the presence of aMPV/C in wild birds in the southeast regions of the U.S.

**Methods:**

In this study, aMPV/C positive oral swabs from American coots (AC) and Canada geese (CG) were passaged three times in the respiratory tract of specific pathogen free (SPF) turkeys and used as aMPV/C P3 virus isolates in subsequent studies.

**Results:**

Wild bird P3 isolates showed similar growth characteristics when compared to virulent aMPV/C in chicken embryo fibroblast ( CEF) cell cultures and their glycoprotein G gene sequence was closely related to the G gene of aMPV/C Colorado reference virus. Three-day-old commercial or SPF turkeys were inoculated oculonasally with wild bird aMPV/C P3 isolates. At 5 and 7 days post-inoculation (DPI), severe clinical signs were observed in both of the AC and CG virus-exposed groups. Viral RNA was detected in tracheal swabs by reverse transcriptase polymerase chain reaction (RT-PCR). In addition, immunohistochemistry showed virus replication in the nasal turbinate and trachea. All virus-exposed turkeys developed positive antibody response by 14 DPI.

**Conclusions:**

Our data demonstrate that aMPV/C wild bird isolates induced typical aMPV/C disease in the domestic turkeys.

## Background

Avian metapneumovirus (aMPV), previously known as turkey rhinotracheitis virus (TRTV), is the cause of severe respiratory infection in turkeys [1-4] and may also be the etiologic agent of “swollen head syndrome (SHS)” of chickens [2]. Originally identified in South Africa in the late 1970s [5], aMPV later spread to Europe, Asia, and South America [4,6-11]. aMPV/C was first isolated in the United States during 1996 in Colorado [12,13] and subsequently outbreaks were reported in Minnesota [14] where the disease has emerged as a major economic problem for the turkey industry. The virus has also spread to other states, such as North Dakota, South Dakota, Iowa, and Wisconsin [15]. aMPV is now considered a major disease threat in both turkeys and chickens in many parts of the world.

aMPV is a member of the genus *Metapneumovirus* in the subfamily *Pneumovirinae* of the family *Paramyxoviridae*[16]. Members of the genus *Metapneumovirus* contain a nonsegmented, single-stranded negative sense RNA genome with the gene order 3’-leader-N-P-M-F-M2-SH-G-L-trailer-5’ [17-22]. The aMPV isolates that exist worldwide are currently classified into four subtypes, namely, subtypes A, B, C, and D. This classification is based on sequence divergence in the attachment (G) glycoprotein and the antigenic differences observed among aMPV strains. The US isolates belong to subtype C [23,24], while the majority of aMPVs isolated in Europe, Asia, and South America belong to the subtype A or B [6,25,26]. aMPV/C was also isolated from farmed ducks in France in 1999 [27] and in live bird market in Korea in 2005 [28]. In 2000, it was reported that aMPVs isolated in France in 1985 belong to subtype D [25].

In the field, aMPV/C leads to mild to severe, rapidly spreading respiratory infection that can adversely affect weight gain and feed conversion. The disease is characterized by depression, coughing, nasal and ocular discharge, and swollen infraorbital sinuses with morbidity of up to 100% [29,30]. Microscopically, inflammatory infiltrates (lymphocytes, macrophages, plasma cells, and heterophils) were detected in nasal turbinate and sinus tissues [31]. Mortality may reach 30% in cases complicated by secondary bacterial infection. Economic losses are due to mortality, a sharp drop in egg production, and increased carcass condemnation rate at slaughter because of air sacculitis [29,31].

The U.S. outbreaks of aMPV/C infection have occurred in a seasonal pattern (spring and fall), and wild migratory birds have been implicated in the spread of the disease [32,33]. In fact, aMPV RNA was isolated from the nasal turbinates of wild sparrows, geese, blue-winged teal, and starlings and shown to share 90 to 95% nucleotide sequence identity with viruses isolated from domestic turkeys [15,32,33]. The role of wild birds in the epidemiology of the disease and transmission of the virus between commercial turkey farms is not clear. Sequence analysis at Southeast Poultry Research Laboratory (SEPRL) of four virus isolates recovered from aMPV/C outbreaks in turkeys shared 95% to 99% nucleotide sequence identity and 97% to 99% predicted amino acid sequence identity with a duck virus isolated from sentinel mallard ducks [34]. These results indicate that aMPV/C isolates from turkeys and ducks share a common source and the viruses from different avian species can cross-infect other birds. In another study the potential role of migratory waterfowl and other wild birds in aMPV/C spread was also examined [35]. Those results showed the presence of antibodies to aMPV/C in American coots, American crows, Canada geese, cattle egrets, and rock pigeons. When oral swabs were assayed by using RT-PCR, aMPV RNA was detected in samples from American coots and Canada geese. Sequence analysis indicated that the wild bird viruses belonged to aMPV/C [35]. The data clearly demonstrated that wild birds can serve as a reservoir of aMPV/C. While the study showed that aMPV/C was able to replicate and induce antibody production in wild birds, the pathogenicity of the aMPV/C isolates for domestic turkeys was not determined [35]. The presence of a potentially pathogenic virus among wild birds could eventually result in the introduction of aMPV/C into poultry flocks in the U.S.

In this study, we determined the pathogenicity of wild bird aMPV/C isolates in domestic turkeys. Biological characteristics of the isolates were studied *in vitro* in cell cultures and by sequence analysis of the G gene. The virulence level and pathogenic properties of the wild bird viruses were demonstrated in experimentally infected SPF and commercial turkeys.

## Results

### aMPV/C-P3 virus isolation from wild birds

Five out of 23 oral swabs from American coots (AC) and 7 out of 27 oral swabs from Canada geese (CG) were found aMPV/C positive by using N and G gene specific RT-PCR (data not shown). The positive swabs were pooled (aMPV/C-AC-P0 and aMPV/C-CG-P0) and subsequently passaged three times in the upper respiratory tract of SPF turkeys, turbinate and tracheal samples were processed as described in materials and methods. Virus stocks from the third turkey passage were designated as P3 viruses and titrated in CEF cultures. The titer of aMPV/C-AC-P3 and aMPV/C-CG-P3 viruses were 10^4.5^ TCID_50_/ml and 10^5^ TCID_50_/ml, respectively. There was no significant difference in growth characteristics and virus yield in CEF cell cultures between the wild bird P3 viruses and the prototype virulent aMPV/C-CO Tr isolate (data not shown).

### The G gene of aMPV/C wild bird isolates

**Figure 1:**
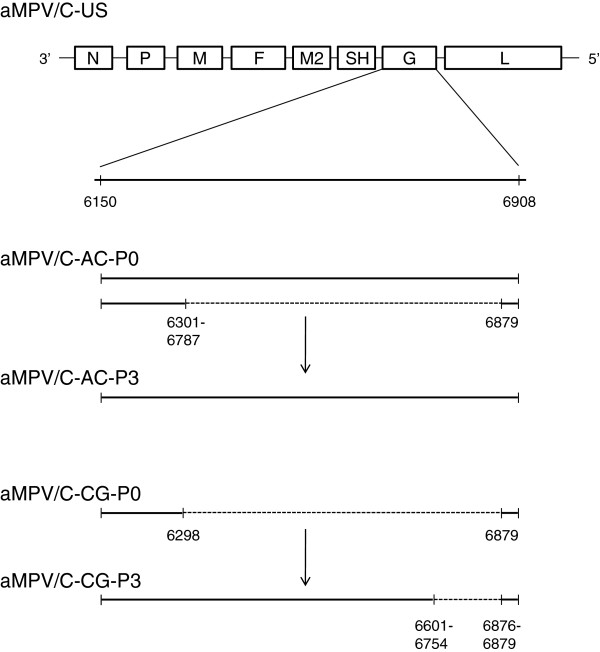
**Schematic representation of aMPV**/**C G gene from pooled American coot** (**AC**) **and Canada goose (CG) samples before (P0)and after three serial turkey passages (P3). **Nucleotide position is indicated by numbers according to GenBank submission of aMPV/C-US strain [GenBank ID: AY579780.1]. Deletions within the G gene are represented by dotted lines and mapped by nucleotide numbers. Multiple nucleotide positions indicate more than one deletion form within the same region of the aMPV/C-AC-P0 and aMPV/C-CG-P3 viruses.

Sequence analysis of the attachment (G) gene from the virus positive AC-P0 pool showed the presence of mixed G gene population that contained both full-length G gene (759 nt) and variants of truncated G genes. The size of the deletion within the truncated G open reading frame (ORF) varied from 93 nt to 579 nt (Figure 1). Only one major G gene fragment was detected within the CG-P0 pool with a deletion of 582 nt (Figure 1). Following three passages in turkeys the AC-P3 virus isolate contained a dominant G gene ORF of 759 nt. This G gene possesses the same length and sequences as that of the Colorado strain reported by Lwamba et al. in 2005 [36]. There was no detectable full-length G gene in the CG-P3 virus; instead a mixed population of shorter G gene ORFs was present with a range of molecules containing deleted segments from 126 nt to 279 nt. Some of these G gene nucleotide deletions were in-frame suggesting the presence of virus variants with truncated but potentially functional G proteins.

### Pathogenicity of wild bird isolates in turkey

**Figure 2:**
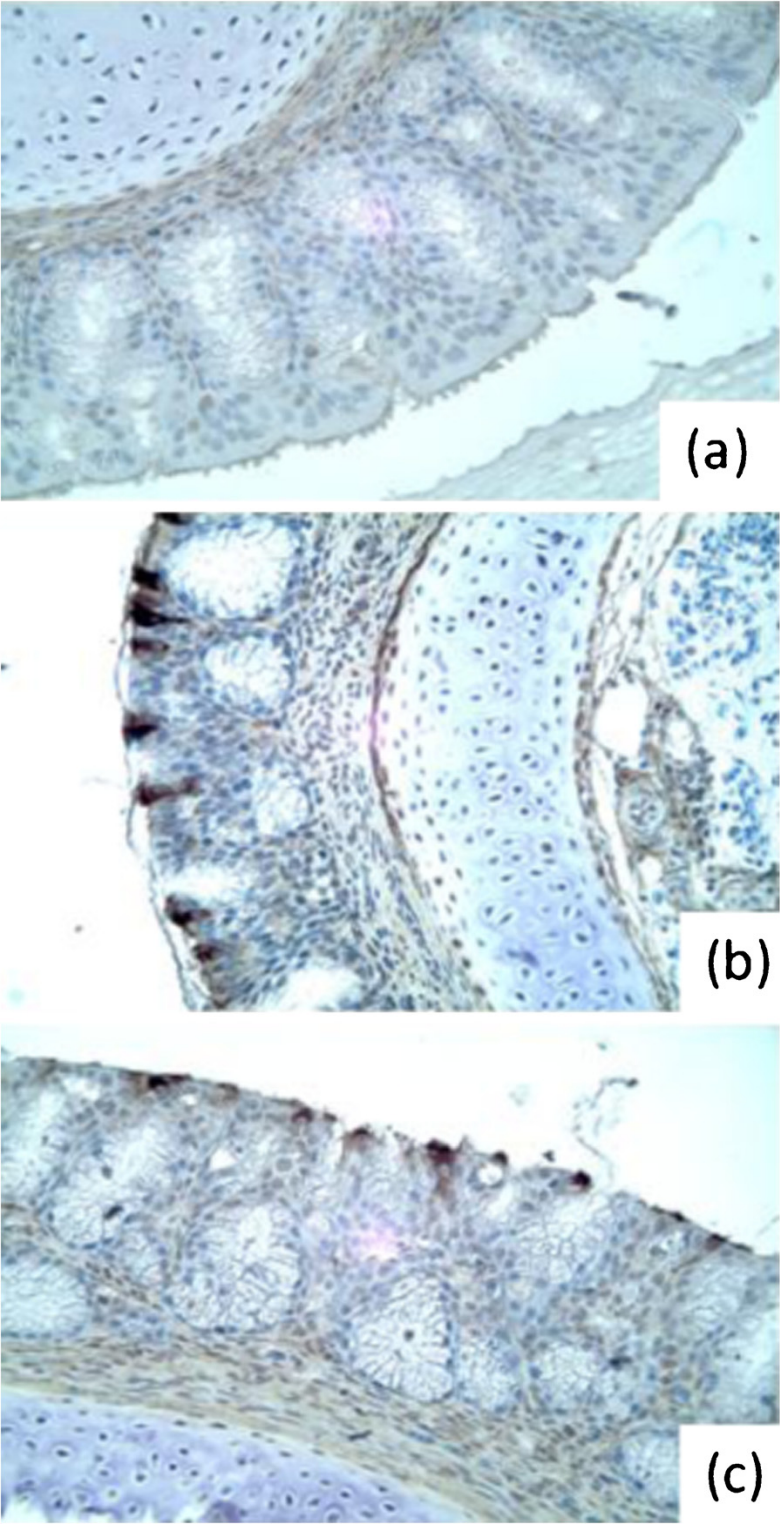
**Detection of aMPV**/**C infected cells in the mucosal layers of nasal turbinate from virus exposed SPF turkeys at 5 DPI by IHC staining. **(**a**) Control untreated turkeys; (**b**) aMPV/C-AC-P3 virus infected turkeys; (**c**) aMPV/C-CG-P3 virus infected turkeys.

In experiment 1, SPF turkeys were inoculated with aMPV/C P3 isolates via ON route. From 5 DPI, the turkey poults exposed to either AC-P3 or CG-P3 viruses exhibited typical clinical signs of aMPV/C disease, showing nasal exudates when squeezed, nasal discharge, or frothy eyes. These clinical signs were still apparent at 7 DPI but were not detectable at later times (Table 1). RT-PCR data to detect viral RNA in tracheal swabs showed that high percentage of inoculated birds became positive at 3 DPI and the number of positives gradually declined by 10 DPI. Microscopic lesions in the turbinate tissues were only observed in a few birds (one positive in each group); however, IHC staining revealed virus replication in the nasal turbinate tissues as early as 5 DPI in 100% of turkeys infected with either AC or CG viruses (Table 1, Figure 2). All birds inoculated with AC or CG viruses developed aMPV/C specific antibody response by 14 DPI, while all sera collected from control birds were negative.

**Table 1:** **Pathogenicity of aMPV**/**C wild bird isolates in SPF turkeys **(**experiment 1**)

**Inoculum**	**aMPV/C RT-PCR**				**Clinical signs**		**Microscopic lesions**		**IHC staining**		**aMPV/C-ELISA**	
**DPI**^**a**^	**3**	**5**	**7**	**10**	**5**	**7**	**5**	**7**	**5**	**7**	**14**	**17**
Untreated	0/11^b^	0/11	0/9	0/7	0/11	0/9	0/2	0/2	0/2	0/2	0/7	0/7
aMPV/C-AC-P3	10/13	6/13	1/10	0/7	11/13	10/10	1/3	0/3	3/3	2/3	7/7	7/7
aMPV/C-CG-P3	6/12	2/12	0/8	1/4	9/12	5/8	0/3	1/3	3/3	2/3	4/4	4/4

^a ^DPI, days post-inoculation.

^b ^Number of positive birds per number in group.

In experiment 2, commercial turkeys were used to study the pathogenic properties of aMPV/C wild bird isolates along with turkeys infected with the challenge virus aMPV/C-CO Tr. Clinical signs, viral RNA detection and seroconversion data following inoculation are summarized in Table 2. Extensive aMPV/C disease specific clinical signs were observed in all groups between 3 and 11 DPI and 100% of the birds seroconverted by 14 DPI. Viral RNA was detectable in turkeys from each group at 3 DPI; however, at later times positives were only observed among the virulent challenge virus inoculated birds. Microscopic lesions were much more abundant in turbinate tissues than in tracheas in each experimental group and IHC also demonstrated a more pronounced virus replication in turbinates from each group when they were compared to those observed in trachea samples at 5 and 7 DPI (Table 3). Overall, both wild bird P3 isolates exhibited very similar pathogenicity in commercial turkeys when aMPV/C disease characteristics were compared to those detected following inoculation with virulent virus aMPV/C-CO Tr.

**Table 2:** Pathogenicity of aMPV/C wild bird isolates in commercial turkeys (experiment 2)

**Inoculum**	**aMPV/C RT-PCR**				**Clinical signs**				**aMPV/C-ELISA**
**DPI**^**a**^	**3**	**5**	**7**	**11**	**3**	**5**	**7**	**11**	**14**
Untreated	0/10^b^	0/10	0/10	0/9	0/23	0/19	0/13	0/9	0/9
aMPV/C-AC-P3	4/10^NS^	0/10^NS^	0/10^NS^	0/10^NS^	11/21^*^	14/18^NS^	13/14^NS^	1/10^NS^	8/8
aMPV/C-CG-P3	8/10^NS^	0/10^NS^	0/10^NS^	0/6^NS^	11/19^**^	14/18^NS^	11/12^NS^	0/6^NS^	5/5
aMPV/C-CO- Tr	8/10	1/10	2/10	2/10	4/24	12/22	12/15	3/10	10/10

^a ^DPI, days post-inoculation.

^b ^Number of positive birds per number in group.

^NS^, statistically not significant difference.

^*^, p<0.01.

^**^, p<0.005.

**Table 3:** Immunohistochemistry (IHC) staining and microscopic lesions in commercial turkeys exposed to aMPV/C wild bird P3 isolates (experiment 2)

**Inoculum**	**Turbinate**				**Trachea**			
	**IHC staining**		**Microscopic lesions**		**IHC staining**		**Microscopic lesions**	
**DPI**^**a**^	**5**	**7**	**5**	**7**	**5**	**7**	**5**	**7**
Untreated	0/4^b^	0/4	0/4	0/4	0/5	0/4	0/5	0/4
aMPV/C-AC-P3	4/4	4/4	2/4	4/4	2/4	1/4	0/4	0/4
aMPV/C-CG-P3	4/4	3/4	2/4	3/4	0/4	0/4	0/4	0/4
aMPV/C-CO-Tr	4/4	5/5	2/4	3/5	3/5	1/5	0/5	0/5

^a ^DPI, days post-inoculation.

^b ^Number of positive birds per number in group.

## Discussion

In the present study, we isolated avian metapneumoviruses from wild birds samples collected in 2000 from Georgia, South Carolina and Arkansas in the U.S. Sequence analysis of virus isolates from oral swabs of American coots and Canada geese confirmed that these viruses belong to subtype C aMPV. Our data clearly demonstrate that both AC and CG wild bird viruses were fully pathogenic in SPF and commercial turkeys. Following experimental inoculation, aMPV/C specific clinical signs (with the exception at 3DPI), virus replication and shedding in the upper respiratory tract were indistinguishable in AC and CG virus infected birds from those observed in group of turkeys inoculated with virulent virus aMPV/C-CO Tr.

Wild birds have been considered as important reservoirs for other avian viruses such as avian influenza and Newcastle disease viruses [37,38]. Previous studies in the U.S. have shown the possible involvements of wild birds in the transmission of aMPV/C viruses [15,32,33,39]. Recent report revealed the presence of aMPV/C in wild birds in the southeast regions of the U.S. [35]. Those data demonstrated that wild birds can serve as a reservoir of subtype C aMPV and may provide a potential mechanism to spread aMPVs to poultry. Here, our results clearly demonstrate the possibility of virus transmission from wild birds to domestic turkeys.

Conventional avian metapneumovirus isolation methods usually include several passages of the virus in avian and mammalian cell cultures. While this technique is convenient and routinely used to isolate virus from field samples it has the potential risk that during serial passages in cell cultures the biological characteristics of the virus, including genome structure and pathogenicity could be altered. In fact, it is well documented that a number of live attenuated aMPV vaccines were produced by passage of virulent virus in cell cultures and these attenuated viruses, which had lost their pathogenicity were subsequently used as protective empirical vaccines [40-43]. It had also been reported that after 15 or more Vero cell passages, the attachment G gene is deleted from the genome of aMPV/C [44]. To avoid possible genomic alterations and/or attenuation following serial virus passage in cell cultures, our studies have focused on the isolation of aMPV/C viruses from wild birds using *in vivo* method. Serial passages of aMPV/C in turkeys proved to be a highly efficient technique to prepare virulent challenge viruses in past studies [45,46]. In our studies, three serial passages of RT-PCR positive wild bird oral swab samples in the respiratory tract of SPF turkeys resulted in the isolation of aMPV/C-P3 viruses without having them propagated in cell cultures. It is important to note that the AC-P3 and CG-P3 isolates exhibited similar growth characteristics *in vitro* in CEF cell cultures when cytopathic effect and virus yields were compared to those observed in cultures infected with virulent aMPV/C-CO Tr virus. These data indicated that using the wild bird P3 isolates in subsequent experiments in turkeys provided true and relevant information about the pathogenic properties of aMPV/C viruses carried by wild birds in nature.

aMPV G gene encodes a major structural glycoprotein that plays important role in viral attachment, pathogenicity and protective immunity [45,47]. The G protein of aMPV isolates exhibits extensive genetic and antigenic variations that are the primary criteria for aMPV subtype classification [7,48,49]. The G gene in aMPV/C isolates exhibits major sequence divergence and the length of the genome reported for different aMPV/C strains isolated in the U.S. is strikingly different [32,36,50,51]. Although recent studies indicated that truncation of the G genes can occur during serial passages of the virus in cell cultures or circulation of the virus in a host in the field [44,52] the biological significance of the G gene length variation is mostly unknown. Our data provide further evidence that the G protein may play an important role in viral pathogenesis in domestic turkey. While the original pooled wild bird samples contained a mixture of virus population with mostly truncated G genes with large deletions, following three passages in turkey the P3 virus stocks contained either full-length G gene (AC-P3) or truncated G genes (CG-P3) with in-frame deletions. It is tempting to speculate that while the G gene may not play important role for aMPV/C replication in wild birds, it is required for efficient virus growth in turkeys. This hypothesis is supported by recent studies, which showed that deletion of the G gene from virulent aMPV/C viruses resulted in attenuation of the virus in turkeys [45,47].

In conclusion, we successfully demonstrated that aMPV/C viruses carried by wild birds in the U.S. possess a potential threat to commercial turkeys. These wild bird viruses are fully virulent and capable of initiating aMPV/C disease outbreaks in susceptible flocks by direct transmission of the virus between wild birds and turkeys. This finding provides further and more definite reason of maintaining high biosecurity standard in the poultry industry.

## Materials and methods

### Viruses and cell cultures

The aMPV/C Colorado strain from turkeys (aMPV/CO/96/C) was obtained from the aMPV repository bank at SEPRL (USDA-ARS, Athens, GA, USA). The virus was propagated in Vero cells (ATCC, Manassas, VA, CCL-81). A virulent challenge virus stock aMPV/C-CO Tr was prepared by three consecutive *in vivo* passages of the aMPV/CO/96/C in turkeys as previously described [45] and was titrated in 1-week-old turkeys for 50% infective dose (ID_50_). Oral swabs from wild birds [American coot (AC) (n=23) and Canada goose (CG)] (n=27) were obtained from SEPRL collection and tested for aMPV/C by reverse transcriptase-polymerase chain reaction (RT-PCR) as described in previous studies [44]. Virus positive swabs from each wild bird were pooled, labeled as P0 (aMPV/C-AC-P0, n=5; aMPV/C-CG-P0, n=7), and inoculated via oculonasal (ON) route to 3-day-old SPF turkeys at a dose of 200 μl inoculum per bird. At 5 days post-inoculation (DPI), turbinate and trachea were pooled and 20% homogenates were prepared with 1x phosphate buffered saline (PBS). Two more passages were repeated in the same manner in 3-day-old SPF turkeys to obtain P3 stock virus of each wild bird isolate (aMPV/C-AC-P3; aMPV/C-CG-P3). To rule out the possibility of the presence of avian influenza virus (AIV) and Newcastle disease virus (NDV) in the P3 isolates, 9-day-old embryonating chicken eggs were inoculated with the P3 stock viruses and after three blind passages hemagglutination assays were performed from the chorioallantoic fluids as described previously [37,45]. Both aMPV/C-AC and aMPV/C-CG P3 isolates were negative to AIV and NDV (data not shown).

Virus titration was performed as previously described [45] with some modification. Briefly, 96-well plates were seeded with SPF chicken embryo fibroblast (CEF) cells and infected with 100 μl of serial 10-fold dilutions of the viruses. After 4–5 days of incubation at 37°C, plates were fixed with 100% ethanol and indirect fluorescence assay was performed using aMPV/C hyperimmune turkey serum. Titers were calculated by the Reed and Muench method [53] and are expressed as 50% tissue culture infective dose (TCID_50_). Virus growth was examined in CEF cell cultures at 0.01 multiplicity of infection (MOI). At 4 days post-infection, the monolayers were harvested and virus was titrated as described above.

### RT-PCR

RNA was extracted from oral or tracheal swabs using Viral RNA Extraction Kit (Qiagen, Valencia, CA) and one-step RT-PCR was performed with a Superscript III one-step RT-PCR Kit (Invitrogen, Carlsbad, CA), following the manufacturer’s protocol. aMPV/C N gene-specific primers were designed based on gene sequence in GenBank [GenBank ID: AM293284] [45]and primers to detect aMPV/C G gene were used as previously described [44]. The sequence of aMPV/C N gene forward primer was 5’-ATGTCTCTTCAGGGGATTC-3’ and reverse primer was 5’-GCATCATTCAGCACACG-3’, the expected size of the amplicon was 650 bp.

### Nucleotide sequencing

aMPV/C G gene specific RT-PCR products were separated in ethidium bromide agarose gel, fragments of expected size were excised and DNA was extracted using a Qiagen MinElute Kit (Qiagen, Valencia, CA). Purified DNA was ligated into the TOPO PCR 2.1 cloning Vector (Invitrogen, Carlsbad, CA) and was used to transform DH5α competent cell (Invitrogen, Carlsbad, CA). Colonies were incubated in LB broth (containing kanamycin) and DNA was prepared using plasmid miniprep purification kit (5 PRIME Inc., Gaithersburg, MD). Sequencing was performed with M13 universal forward and reverse primers using the Applied Biosystems-PRISM fluorescent big dye sequencing kit and the ABI 3730 automated DNA sequencer (ABI, Foster City, CA). The sequence obtained from each colony was compared and analyzed with those in the GenBank using the BLAST algorithms and DNASTAR program (Madison, WI).

### Animal experimental design

SPF turkeys were obtained from the SEPRL flocks, aMPV/C antibody-free hybrid commercial turkeys were received from Prestige farm (Charlotte, NC). The birds were housed in Horsfal isolators (Federal Designs, Inc., Comer, GA) with ad libitum access to feed and water. Sera from one-day-old turkeys (n=10) were examined by aMPV/C-specific enzyme-linked immunosorbent assay (ELISA) to ensure the absence of anti-aMPV/C antibodies. All experimental procedures in turkeys were approved by the SEPRL Institutional Animal Care and Use Committee.

In experiment 1, four-day-old SPF turkeys were divided into three groups of 15 birds. Birds in group 1 were untreated and served as negative controls. Birds in groups 2 and 3 were inoculated via oronasal (ON) route with 200 μl of aMPV/C-AC-P3 (10^4.5^ TCID_50_/ml) and aMPV/C-CG-P3 (10^5^ TCID_50_/ml), respectively. At 3, 5, 7 and 10 DPI, tracheal swabs were collected (from all available in each group, n=4-13) and clinical signs for aMPV disease were observed as described previously [45]. Turkeys were considered clinically positive if showing nasal exudates when squeezed, nasal discharge or frothy eyes. Turbinate and trachea tissues for histopathology and immunohistochemistry (IHC) were randomly collected at 5 and 7 DPI (n=2 from controls and n=3 from inoculated) and serum to detect virus specific antibodies was collected at 14 DPI (n=7 from control and AC group; n=4 from CG group).

In experiment 2, three-day-old aMPV/C antibody free commercial turkeys were randomly divided into four groups of 25 birds and inoculated via ON routes as described in experiment 1. Birds in group 1 were untreated, birds in group 2 and 3 received aMPV/C-AC-P3 and aMPV/C-CG-P3, respectively. Each bird in group 4 was infected with 200 μl aMPV/C-CO Tr (10^4.6^ ID_50_/ml) via ON route as a positive control. At 3, 5, 7 and 11 DPI, tracheal swabs were collected (n=10) and the birds were observed for recording aMPV clinical signs. Turbinate and trachea tissues for histopathology and IHC (n=4-5) were randomly collected at 5 and 7 DPI and serum samples (n=5-9) for aMPV/C specific ELISA were collected at 14 DPI.

### Histopathology and immunohistochemistry

For histopathology, turbinate and trachea tissues were fixed in formalin and embedded in paraffin. Sections were sliced into 4 μm and stained with haematoxylin and eosin (HE). All HE stained sections were examined by light microscope. Turkeys were considered positive if extensive lymphoid cell infiltrations were observed in the mucosal layer of turbinate or trachea as shown in previous reports [31,54,55]. Virus infected cells were detected by IHC staining as previously described with some modifications [56,57]. Briefly, paraffin embedded sections (4 μm) of turbinate or trachea tissues were deparaffinized, dehydrated followed by antigen retrieval with citrate buffer (Biogenex, San Ramon, CA). Rabbit anti-aMPV/C hyperimmune serum (kindly provided by Dr. K. Nagaraja of University of Minnesota) was used as primary antibody in1:1000 dilution. For secondary antibody, goat anti-rabbit IgG conjugated with horseradish peroxidase (HRP) (KPL, Gaithersburg, MD) was used at dilution of 1:250. The signal was developed with 3'-diaminobenzidine (DAB) Kit (Vector Laboratory, Burlingame, CA) following manufacturer’s instruction.

### ELISA

Anti-aMPV/C antibody response of turkeys was determined by an aMPV/C-specific IgG ELISA as previously described [45]. Briefly, turkey sera were diluted (1:100) and added to 96-well plates in triplicates, which were coated with sucrose gradient purified aMPV/CO/96/C virus as antigen. HRP conjugated goat anti-turkey IgG antibody (KPL, Gaithersburg, MD) was added as secondary antibody. A luminal-based chemiluminescent substrate (Lumiglo substrate kit; KPL, Gaithersburg, MD) was used as a developing agent. The chemiluminescence relative light unit (RLU) was measured on a Biotek Synergy HT Microtiter Plate Luminometer (Biotek Instruments, Winooski, VT). Test sera were considered positive when the mean RLU value was higher than the mean RLU plus 2 × standard deviation of the negative turkey serum pool.

### Statistics

The statistical analyses were done using the Fisher’s exact test (SigmaStat 2.0.3, SPSS Inc, Chicago, IL) in Experiment 2, where groups of turkeys inoculated with aMPV/C-AC-P3 or aMPV/C-CG-P3 viruses were compared to those infected with virulent aMPV/C-CO-Tr challenge virus.

## Abbreviations

AC: American coot; AIV: Avian influenza virus; aMPV/C: avian metapneumovirus subtype C; CEF: Chicken embryo fibroblast; CG: Canada goose; DAB: Diaminobenzidine; DPI: Days post-inoculation; ELISA: Enzyme-linked immunosorbent assay; HRP: Horseradish peroxidase; ID_50_: 50% infective dose; IHC: Immunohistochemistry; MOI: Multiplicity of infection; NDV: Newcastle disease virus; ON: Oculonasal; ORF: Open reading frame; PBS: Phosphate buffer saline; RLU: Relative light unit; RNA: Ribonucleic acid; RT-PCR: Reverse transcriptase polymerase chain reaction; SEPRL: Southeast Poultry Research Laboratory; SHS: Swollen head syndrome; SPF: Specific pathogen free; TCID_50_: 50% tissue culture infective dose; TRTV: Turkey rhinotracheitis virus.

## Competing interests

The authors declare that they have no competing interests.

## Authors’ contributions

RMC conceived and planned the study, performed the animal experiments, and co-wrote the paper. QY prepared the wild type virus stocks and co-wrote the paper. LZ coordinated the studies, supervised the animal experiments and co-wrote the paper. All authors read and approved the final manuscript.
